# Therapeutic goals and treatment response evaluation in moderate to severe psoriasis: an experts opinion document

**DOI:** 10.1080/07853890.2021.1986637

**Published:** 2021-10-03

**Authors:** Isabel Belinchón Romero, Esteban Dauden, Carlos Ferrándiz Foraster, Álvaro González-Cantero, Jose Manuel Carrascosa Carrillo

**Affiliations:** a Department of Dermatology, Hospital General Universitario de Alicante-ISABIAL-UMH, Alicante, Spain; b Department of Dermatology, Hospital Universitario de La Princesa, Instituto de Investigación Sanitaria La Princesa (IIS-IP), Madrid, Spain; c Department of Dermatology, Hospital Universitari Germans Trias i Pujol, Badalona, e IGTP, Universitat Autònoma de Barcelona, Barcelona, Spain; d Department of Dermatology, Hospital Universitario Ramón y Cajal, Madrid, Spain

**Keywords:** Psoriasis, therapeutic objective, treatment response, systematic literature review, Delphi

## Abstract

**Objective:**

To critically analyse and define therapeutic objectives, response to treatment evaluation and related decisions in psoriasis.

**Methods:**

Expert consensus meetings, a systematic and narrative reviews and a collaborative Delphi procedure were carried out. A steering committee from the Spanish Group of Psoriasis was established who based on the reviews generated a set of related statements. Subsequently, a group of 40 experts tested their agreement with the statements, through 3 Delphi rounds.

**Results:**

We found a great variability in clinical guidelines regarding to the definition of treatment goal and the response. In general, treatment failure was considered if a PASI50 is not achieved. The panel of experts agreed on (1) clearly differentiate between ideal and a realistic goals when establishing the therapeutic goal in moderate to severe psoriasis; (2) treatment goals should be in general established regardless of the type of drug for psoriasis; (3) treatment failure if PASI75 response is not reached; (4) an absolute PASI is in general preferred to the rate of PASI improvement from baseline; (5) disease characteristics, patients and physicians opinions/needs and treatment adherence influence treatment goals.

**Conclusions:**

A clear treatment decision making framework is vital to improve management of psoriasis.

**KEY MESSAGES**
- Psoriasis characteristics, patients and physicians opinions/needs and treatment adherence influence treatment goals.
- Different disease indexes could be used to assess treatment response but absolute PASI is preferred
- In general psoriasis treatment failure should be considered if PASI75 response is not reached

## Introduction

Psoriasis is a chronic, immune-mediated inflammatory skin disease affecting 2–3% of the Caucasian population in western countries [1,2]. It is associated with increased incidence and prevalence of certain comorbidities and significant impact on patients’ quality of life [3].

In recent years, new treatments and treatment strategies have emerged and changed the paradigm of psoriasis, especially for patients with moderate to severe disease [4]. Among new treatments, biologics provide targeted inhibition of immune-mediated pathways involving specific cytokines, such as tumour necrosis factor (TNF), interleukin (IL)-17, or IL-23. Their clinical efficacy have been extensively demonstrated in randomised clinical trials (RCTs) and observational studies that have also depicted superiority compared to traditional systemic drugs like methotrexate [5].

On the other hand, the role of patients in the management of the psoriasis have also changed. Nowadays, patients are more active in the management of the disease, and their opinion is taking into account in the decision making [6]. Besides, currently, many studies include not only objective variables to assess efficacy like the Psoriasis Area and Severity Index (PASI), the Psoriasis Global Assessment (PGA) or the Body Surface Area (BSA) [7], but also patient-reported outcomes (PROs) including quality of life because objective assessment of the disease does not always strictly correspond to the patient’s subjective perception [8–10].

However, it has been shown that psoriasis is often less than optimally treated, especially in patients with moderate to severe psoriasis. According to different studies, overall, patient satisfaction with existing therapies remains modest [11], and many of them present high disease burden despite treatments [12]. Different factors might explain this results [13,14]. One of them is the lack of a generally accepted consensus definition of either treatment goals or treatment success/failure (and subsequent treatment modifications) [15]. As highly effective new treatments and treatment strategies are available and patient’s involvement is increasing, this turns out vital.

Taking into account all of the exposed above, we proposed this project to explore current definitions of therapeutic objectives, response to treatment and related clinical decisions in moderate to severe psoriasis, in order to propose an appropriate (and agreed) treatment decision making framework. For this purpose, we developed a systematic literature review (SLR), analysed main clinical guidelines/consensus documents and tested different statements through a Delphi procedure.

## Methods

**Figure 1.**
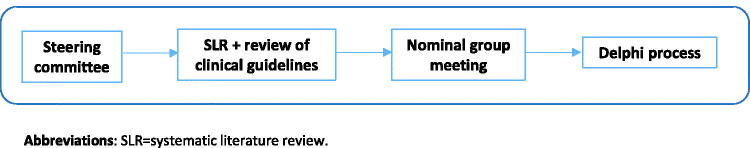
Project methodology flowchart. Abbreviation. SLR: systematic literature review.

This study was conducted in accordance with Good Clinical Practice guidelines and the current version of the revised World Medical Association’s Declaration of Helsinki. This study did not require formal ethical approval because this was not a study involving humans. This was a qualitative study based on expert opinion. See Figure 1.

### Steering committee establishment

A group of 5 experts in psoriasis was established. The criteria for the selection were the following ones: (1) Dermatologist; (2) Specialised in psoriasis with demonstrated clinical experience; (3) Clinical experience ≥8 years and/or ≥5 publications; (4) Participation in clinical trials or other observational projects in psoriasis; (5) Members of the psoriasis working group of the Spanish Academy of Dermatology and Venereology (AEDV).

They were responsible of: (1) The selection and invitation of the expert panel; (2) Protocol of the systematic literature review (SLR) and the identification of the main psoriasis clinical guidelines; (3) Generation of clinical statements and modifications after each Delphi round; (4) Definition of the consensus levels and agreement on methodology; (5) Interpretation of the results from the Delphi rounds; (6) final edition of the publication.

### SLR and narrative review of clinical guidelines/consensus documents

The aim of the SLR was to assess PASI 100 response of topic and systemic therapies in psoriasis (will be published in a separate article). Along with the SLR, a narrative literature review was conducted in which several national and international clinical guidelines/consensus documents were analysed in order to collect their definitions of treatment goal and treatment success/failure, objective measures and treatment decisions/modifications [16–25].

### Nominal group meeting

The steering committee analysed and discussed the results of the SLR and of the national and international clinical guidelines/consensus documents. Based on them and their experience, they generated several clinical statements that were organised in 2 main topics. The first one related to therapeutic goal, and the second one on treatment response evaluation and related clinical decisions (see supplementary material). In both cases, different definitions, limits, objective measures and associated factors were examined and tested in order to be accurate with the agreements (see supplementary material). The steering committee also defined the Delphi methodology and consensus levels.

### Delphi process

The clinical statements were subsequently submitted to a Delphi process. For this purpose, apart from the steering committee, 35 more expert dermatologists members of the psoriasis working group of the AEDV that were invited to participate in an on-line 3 Delphi rounds. In order to ensure appropriate methodological rigour, consistent agreement and consistent disagreement (consensus) required fulfilment of ≥ 2 of the following 3 criteria. Criterion 1: Mean is 8 or 9, or 1 or 2 and standard deviation (SD) <2. Criterion 2: The median is 8 or 9, Q3 > 7, and Interquartile range (IQR) <2, or the median is 1 or 2, Q3 < 3, and IQR <2. Criterion 3: If ≥70% of experts vote 8 or 9 (consistent agreement), or if ≥70% vote 1 or 2 (consistent disagreement). After each round, a facilitator provided an anonymous summary of the experts’ forecasts from the previous round. The results of the first and second Delphi rounds were analysed by the steering group. Clinical statements that achieved consensus were not voted on a subsequent round. Those that did not, were reassessed by the steering committee and, if appropriate, re-edited and voted on in a second round. After the first second round, the inclusion of new clinical statements was allowed.

## Results

### Delphi process

A total of 30 statements were voted in the first Delhi round of which 9 achieved consensus (agreement) and were not included in the next round. Six were modified, and 37 new statements were also incorporated into the second Delphi round. After this round, 18 statements reached consensus (all but 1 on the agreement). The remaining statements went to the third Delhi round in which 9 more achieved consensus (all but 2 on the agreement). See supplementary material for more details.

Here, we present the main results of the Delphi process along with those from the reviewed clinical guidelines/consensus documents in the management of moderate to severe psoriasis patients.

### Therapeutic goal

The definition and approach of moderate to severe psoriasis treatment goal varies depending on the clinical guideline/consensus document (Table 1). In summary, these initiatives formulate (1) a set of overarching principles (a final therapeutic goal/s and their characteristics); (2) a context (different factors that influence and modulate the therapeutic goal/s) and measures to objectively describe/analyse the treatment goals.

**Table 1.** Treatment goals definitions for patients with moderate to severe psoriasis according to clinical guidelines/consensus documents.

#	Document	Definition
1	French guideline of systemic therapy [23]	-Several factors should be taken into account when establishing treatment goals for systemic therapy: Disease severity (measured as either an absolute value or by comparison with baseline values) Presence of PsA or any other comorbidities Disease impact on the physical, psychological and social well-being of the patient The risk–benefit ratio of continuous systemic treatment Patient’s point of view and level of satisfaction
2	Australian consensus of treatment goal [26]	-Not clearly stated -Based on treatment response and failure
3	European consensus of treatment goal [15]	-The ultimate goal of any psoriasis treatment is to achieve complete clearance of skin symptoms -It has to be based upon the results achievable with available treatments as indicated by the results of RCTs and the outcomes observed in clinical practice -It needs a minimal degree of improvement (principle of the lowest hurdle) and the drug-specific evaluation time points
4	Spanish consensus of systemic therapy [18,25]	-The ideal outcome is to achieve: PASI 90, PGA ≤ 1, or alternatively a minimal and controllable localised involvement with topical treatments (PGA ≤ 2 and PASI < 5), DLQI ≤ 1, prolonged remissions without loss of efficacy, no worsening of comorbidities -Criteria for an appropriate response initially and in the long term (more than 6 months), ≥1 of: PASI 75, PASI < 5, PGA ≤ 1, DLQI < 5 -Criteria for the minimum efficacy required: PASI 50, PASI <50 if the patient is satisfied with the result, DLQI < 5
5	European guideline of systemic therapy [20,21]	-Not specifically stated -PGA 0/1 (i.e. “clear”/“almost clear”) in the induction and in long-term is considered as critical
6	European consensus of apremilast / secukinumab [22]	-It uses the same criteria of the European consensus of systemic therapy [20,21]
7	Italian guideline of systemic therapy [24]	-Treatment goals should be agreed with the patient based on informed discussion PASI 75 PASI 90 There is a need to define the minimum absolute PASI (i.e. <1 or 2) DLQI < 5
8	British guideline of systemic therapy [27]	-Tailor the choice of agent to the needs of the person and take into account the following factors Psoriasis factors: Goal of therapy (e.g. PGA of clear or nearly clear) Disease phenotype and pattern of activity Disease severity and impact The presence of psoriatic arthritis (in consultation with an adult or paediatric rheumatologist) Other factors: Person’s age Past or current comorbid conditions (e.g. inflammatory bowel disease, cardiovascular disease) Conception plans Body weight Person’s views and any stated preference on administration route or frequency Adherence
9	American consensus of fototherapy and systemic therapy [16,17]	-Not specifically stated

Abbreviations. PsA: psoriatic arthritis; PASI: Psoriasis Area and Severity Index; BSA: Body Surface Area; DLQI: dermatology life quality index; RCT: randomised controlled trial; PGA: Physician’s Global Assessment.

Some of clinical guideline/consensus documents define the overarching principles regarding to treatment goal as an ideal clinical situation in which psoriasis disease is not present, for example, “*The ultimate goal of any psoriasis treatment is to achieve complete clearance of skin symptoms*” [15,18]. A Spanish consensus also comprises a time frame and the possibility of an additional and less strict therapeutic goal in these overarching principles as follows “*…to maintain in the long term complete or almost complete clearing or, failing that, a minimal and localized affected area*…” [18,25]. These documents also propose measures (and different cut-offs) like PASI 75 [24], PASI 90 [24,25], absolute PASI score <5 [18], BSA [18], PGA = 0 [20,21], PGA ≤ 1 [18,24,25], PGA ≤ 2 [18,24] or DLQI < 5 [24], to objectively define treatment goals. Besides, most of these clinical guideline/consensus documents estate that treatment goal should be established taking into account different disease, patients, treatments and other local factors (the context), like disease severity, patients preferences, no worsening of comorbidities or the risk–benefit ratio of continuous systemic treatment [15,23,25,27].

On the other hand, connected to the evaluation of the treatment response, the European consensus of psoriasis treatment goal [15] depicts that it needs a minimal degree of improvement (principle of the lowest hurdle) and drug-specific evaluation time points [15]. The same way, the Spanish consensus generated a set of criteria for an appropriate response at the start of treatment and in the long term (see Table 1) [25].

All of these questions were tested in the Delphi process (Table 2 and supplementary material). The panel of experts agreed on clearly differentiate between ideal and a realistic goals when establishing the therapeutic goal in moderate to severe psoriasis. And, based on this, several criteria to define them were also agreed. For ideal goals, 2 types of criteria were accepted. Those reflecting a complete resolution of the disease like the absence of psoriasis-related symptoms, impact and in terms of measures a PASI 100 response, absolute PASI score 0 or skin clearance, but also those that might reflect “an almost resolution” of the disease like PASI 90 and PASI score ≤1 or ≤2 were considered. Interestingly, PASI 90 and PASI score ≤2 were included in the realistic goals as well along with a PASI score ≤3. Besides, in this group of goals, and just for some patients, a PASI 75 or PASI score ≤5 could be considered a realistic goal.

**Table 2.** Main messages from the statements that achieved consensus in the Delphi process regarding to therapeutic goals in moderate to severe psoriasis.

#	Consensus (on the agreement)
1	The therapeutic goal should be:
1.1	Individualized
1.2	Adapted to the characteristics of the disease
1.3	Adapted to the characteristics of the patient
1.4	Established in general regardless of the type of drug for psoriasis
2	When establishing the therapeutic goal in moderate to severe psoriasis, it is advisable to differentiate between an ideal goal and a realistic goal
3	Ideal goals should include:
3.1	PASI 100 response, absolute PASI score 0 or skin clearance
3.2	Absence of psoriasis-related symptoms
3.3	Absence of impact of psoriasis on patient’s psychological, emotional, social and occupational domains
3.4	Achieving PASI 90 response
3.5	Achieving an absolute PASI score ≤1
3.6	Achieving an absolute PASI score ≤2
4	Realistic goals should include:
4.1	Achieving PASI 90 response
4.2	In general, realistic goals should include achieving PASI 90 response, but sometimes, achieving PASI 75 response may also be realistic
4.3	Achieving an absolute PASI score ≤2
4.4	Achieving an absolute PASI score ≤3
4.5	In general, realistic goals should include achieving an absolute PASI score ≤3, but sometimes, achieving an absolute PASI score ≤5 might also be a realistic goal
4.6	In general, realistic goals should include achieving an absolute PASI score ≤3, but, with some drugs for psoriasis, achieving a PASI score ≤1 might also be possible

Abbreviation. PASI: Psoriasis Area and Severity Index.

We would like to point out that regarding to the context, similar factors as those shown in clinical guidelines/consensus documents were agreed, but the experts specifically added that in general, treatment goals should be established regardless of the type of drug for psoriasis.

However, the experts did not differentiate treatment goals depending on the treatment time point. Treatment goals are the same along the time.

### Response to treatment and related clinical decisions

We next analyse response to treatment definitions across the clinical guidelines/consensus documents. Many of them stablish criteria/definitions for 2 (considered) different phases, the induction and the maintenance phases [18,20,21,25,26], see Table 3. In general, treatment failure is defined when an improvement of PASI of ≥50% is not achieved. The same way, if an improvement of PASI of ≥ 50% but <75% is achieved but a DLQI < 5 not, then this situation is also considered as treatment failure.

**Table 3.** Definitions of treatment response and/or failure and treatment modifications for patients with moderate to severe psoriasis according to clinical guidelines/consensus documents.

#	Document	Definition
1	French guideline of systemic therapy [23]	-Treatment response is considered as adequate if PASI 75 from baseline, or PASI 50 and a DLQI score ≤5 -Primary treatment failure as not achieving a PASI 50 response
2	Australian consensus of treatment goal [26]	-Treatment failure after induction phase: If at the end an improvement of PASI of ≥50% as compared to disease severity at the time of treatment initiation has not been achieved If at the end an improvement of PASI of ≥50% but <75% as compared to disease severity at the time of treatment initiation has been achieved (intermediate response), but DLQI ≤ 5 has not been achieved -Treatment success during maintenance phase: If an improvement of PASI of ≥75% as compared to disease severity at the time of treatment initiation has been achieved -Treatment failure during maintenance treatment: If an improvement of PASI of ≥50% as compared to disease severity at the time of treatment initiation has not been achieved If an improvement of PASI of ≥50% but <75% as compared to disease severity at the time of treatment initiation can be maintained, but DLQI <5 has not been achieved -When treatment failure modification of the treatment regimen is recommended
3	European guideline of systemic therapy [20,21]	-During induction and maintenance phase: Treatment can be continued if PASI 75 is achieved Treatment regimen should be modified if improvement of PASI 50 is not achieved If PASI response is between 50 and75%, therapy should be modified if the DLQI is >5 but can be continued if the DLQI is ≤5
4	Spanish consensus of systemic therapy [18,25]	-Primary therapeutic failure: Lack of response or primary failure occurs when at the end of the induction phase a score is equal to or greater than those constituting the criteria for moderate-to-severe psoriasis Lack of response or primary failure occurs when there is no adequate response by the end of the induction phase, according to both the physician and the patient Lack of response or primary failure occurs when a decrease in 50% from the baseline PASI score has not been achieved (or this degree of response has been lost) after the induction phase
5	British guideline of systemic therapy [27]	-Review response to biologic therapy by taking into account: Psoriasis disease severity compared with baseline (e.g. PASI) Agreed treatment goal Control of PsA disease activity and/or IBD Impact of psoriasis on the person’s physical, psychological and social functioning Benefits vs. the risks of continued treatment Views of the person undergoing treatment (and their family or carers, where appropriate) Adherence to the treatment -Assess whether the minimal response criteria have been met, as defined by: ≥50% reduction in baseline disease severity (e.g. PASI 50 response, or percentage BSA) and clinically relevant improvement in physical, psychological or social functioning (e.g. ≥4-point)

Abbreviations. PsA: psoriatic arthritis; IBD: inflammatory bowel disease; PASI: Psoriasis Area and Severity Index; BSA: Body Surface Area; DLQI: dermatology life quality index; PGA: Physician’s Global Assessment.

More specifically the Spanish consensus of systemic therapy [18,25] states that definitions for treatment failure can be established based on an absolute PASI score or response, PGA, and a quality-of-life score, either individually or in combination. Lack of response or primary failure occurs when there is no adequate response by the end of the induction phase, according to both the physician and the patient. Similarly, the British guideline of systemic therapy [27], depicts a list of factors (related to patients, disease and treatment) that should be analyse when reviewing the response to biologic therapy. They also recommend to assess whether the pre-defined minimal response criteria have been met (see Table 3). All of the clinical guidelines/consensus documents agree on modifications of treatment when the response rate is not reached. The modifications include different strategies/actions like dose increase, combinations or biologic switch.

The main results of the Delphi regarding to this section are shown in Table 4 (more details are exposed in the supplementary material). In this case, a part of the discussions and statements were related to the assessment of response to treatment with the absolute PASI score or the PASI response (change from the initiation of the treatment). Based on the Delphi results, in general, in daily practice, the absolute PASI score was preferred to the rate of PASI improvement. However, the experts also consider that the last one sometimes can provide additional information to the absolute PASI score. The same way, when complete clear skin is assessed, it was agreed that either the PASI 100 response or the PASI absolute score 0 can be used, but the absolute score was considered more appropriated.

**Table 4.** Main messages from the statements that achieved consensus regarding to treatment response and/or failure and treatment modifications in moderate to severe psoriasis.

#	Consensus (on the agreement)
1	Response to treatment could be assessed by absolute PASI
2	Sometimes, the rate of PASI improvement from baseline, complements the information from the absolute PASI and is useful to assess response to treatment
3	In general, in daily practice, absolute PASI is preferred to the rate of PASI improvement from baseline to assess response to treatment
4	The absolute PASI score 0 is the most appropriate measure to assess skin clearance
5	In daily practice, when complete clear skin is selected to assess response to treatment, the PASI 100 response or the PASI absolute score 0 can be used
6	In daily practice, if PASI 75 response is not reached with a treatment (recommended doses), a treatment change would be justified even if it implies a cost increase
7	In daily practice, if an absolute PASI score ≤3 is not achieved with a treatment (recommended doses), combination therapy would be justified
8	In daily practice, if an absolute PASI score ≤5 is not achieved with a treatment (recommended doses), it would be justified: Treatment change, even if it implies a cost increase Combination therapy
9	In daily practice, if the realistic goal is not achieved with a treatment (recommended doses), a change of treatment strategy it would be justified including: Treatment change Combination therapy
10	In daily practice, if the ideal goal is reached with a treatment (recommended doses), dose reduction (dose down-titration and/or increasing the interval between doses) could be included as a change of treatment strategy
11	In daily practice, if the realistic goal is achieved with a treatment (recommended doses), dose reduction (dose down-titration and/or increasing the interval between doses) could be included as a change of treatment strategy
#	Consensus (on the disagreement)
12	The rate of PASI improvement from baseline is preferred to absolute PASI to assess response to treatment
13	In daily practice, if PASI 100 response is not achieved with a treatment (recommended doses), dose changes would be justified (treatment intensification)
14	In daily practice, if an absolute PASI score ≤1 is not achieved with a treatment (recommended doses), a treatment change would be justified even if it implies a cost increase

Abbreviations. PASI: Psoriasis Area and Severity Index.

Following, when evaluating treatment response, according to this project experts, treatment failure in daily practice was if PASI 75 response, or an absolute PASI score ≤3 or 5 were not achieved with a treatment following recommended doses (Table 4). The attitude in case of treatment failure (type of treatment modification) varied depending on the outcome. More specifically, if PASI 75 response is not reached with a treatment, the experts agreed that a treatment change would be justified even if it implies a cost increase. There was a trend towards to consider as well treatment combinations if PASI 75 response is not achieved. On the other hand, if an absolute PASI score ≤3 is not achieved, the experts only agreed on the use of combined therapy as a treatment modification. However, when an absolute PASI score ≤5 is not achieved, a treatment change even if it implies a cost increase and treatment combinations are options. In general, treatment intensifications (dose increases) were not considered in case of treatment failure in patients with moderate to severe psoriasis.

We then tested treatment failure from the treatment goal perspective (Table 4). We formulated the same statements asking what attitude was appropriate if the realistic goal was not achieved. The experts agreed on a change of treatment strategy including treatment modifications and combinations.

Afterwards, we asked about the treatment decision if the ideal or realistic goal was achieved in daily practice. In both cases, dose reductions (dose down-titration and/or increasing the interval between doses) could be considered as a change of treatment strategy. There was no agreement on stopping the treatment or making a change of treatment strategy.

## Discussion

In this study we have critically analysed therapeutic goal definitions and treatment response evaluation through a revision of main clinical guidelines/consensus documents and through a Delphi process within a group of experts.

The treatment options for patients with moderate to severe psoriasis have expanded greatly over the past decade. Moreover the results of RCTs and clinical practice indicate that the efficacy in many cases is outstanding [4,5]. However, it has been shown that psoriasis treatment might be sub-optimal in many cases [11,12]. Published studies have depicted that psoriasis patients do not receive the appropriated care that is required to clear their skin symptoms and to improve their quality of life [11,12]. Several factors might explain this situation, but the lack of well-defined treatment goals with an integrated demand for action could be definitely contributing to this situation [15].

We design this project to analyse current definitions and recommendations regarding to treatment goals and response evaluation and to provide guide when establishing them in daily practice. For this purpose, we first review national and international clinical guidelines/consensus documents. And afterwards, we generated several clinical statements to deeply explore the opinion and attitudes of a group of expert dermatologists in this regard. Simple definitions and clinical recommendations are usually more easily implemented in daily practice but might not cover or be applied to all patients with heterogeneous diseases like psoriasis. Therefore, the statements were conscientiously generated looking for a balance between simplicity (clarity and applicability) and the coverage of a wide range of psoriasis clinical profiles. The same way, we included different objective measures like PASI or PGA and clinical scenarios trying to stablish possible general preferences but also including flexibility. As a consequence more than sixty statements were tested. Additionally, we would like to comment that the pre-established agreement level is extremely strict. This reinforce the validity of those which reached agreement.

Regarding to treatment goals, first, many clinical guidelines or consensus documents have proposed, as overarching principle, that psoriasis ultimate or ideal goal is to achieve complete clearance and to maintain it in the long term [16–24]. However, it is also recognised that this would not be realistic in many cases as data from RCTs and clinical practice show that not all patients respond appropriately [4,28]. In fact, the Spanish consensus of systemic therapy includes as a possible goal in some cases an almost complete clearing or, failing that, a minimal and localised affected area, providing also criteria for the minimum efficacy required [18,25]. Therefore, as it was also agreed in the Delphi, ideal and realistic goals were clearly differentiated and defined. According to the Delphi experts, ideal goals should always be considered as “the goal” and the first option. But for many patients realistic goals will be the drivers of therapeutic decisions. For ideal objectives, a set of objective measures (e.g. absolute PASI score 0) and clinical variables (e.g. an absence of psoriasis-related symptoms) were included in an effort to combine objective data but also patients experience and opinion. Besides, different types of measures were considered appropriated like PASI response, absolute PASI score or skin clearance. And connected to the cut-offs, the experts agreed to include those reflecting a complete resolution of the disease (0 value) but also others that reflect an “almost resolution” like an absolute PASI score ≤1 or 2. From a practical point of view, it could assumed that an absolute PASI score ≤2 in the absence of psoriasis symptoms and impact on the patient (the real centre of the treatment of psoriasis) as a situation close to a complete clearance, and therefore an ideal clinical scenario.

Remarkably, both, PASI 90 response and an absolute PASI score ≤2, were a part of the ideal and realistic goals in our Delphi. This probably reflects the clinical heterogeneity of psoriasis disease and the factors that influence and modulate each individual patient (the context). For some patients that present more severe disease, comorbidity or intolerance problems, a PASI 90 response would be ideal (though not realistic), but for others this goal could perfectly be attainable. Following the same principle, although the limits of a realistic goal were established in a PASI 75 response and an absolute PASI score ≤3, it was agreed that in some specific cases, achieving a PASI 75 response or an absolute PASI score ≤5 might also be a realistic goal.

It is widely accepted (as well as in our Delphi) that treatment goal should take into account patients, disease, treatments characteristics and local medical environment. We would like to point out that regarding to the context, in our project the experts specifically added that in general, treatment goals should be established regardless of the type of drug for psoriasis. Although psoriasis drugs present different efficacy and safety profiles, these are contextual factors not the main drivers of the therapeutic goal.

On the other hand, we also examined response to treatment evaluation (success and/or failure) and related clinical decisions. The definition of treatment failure was very consistent across the guidelines clinical guidelines/consensus documents, and the criteria was if an improvement of PASI of ≥50% is not achieved, in both, induction and maintenance phase. Most of these documents also accepted a lower level of improvement providing a good quality of life, and at least two of them incorporated some other factors to take into account like patients opinion, or the adherence to treatment [18,27]. All agree on modifications of treatment when the response rate is not reached. In our Delphi, it was first agreed that the rate of PASI improvement from baseline, complements the information from the absolute PASI and is useful to assess response to treatment reflecting that both can be used but the absolute PASI is preferred. Regarding to the cut-offs to define treatment failure, the experts agreed to established them if a PASI 75 response or an absolute PASI score ≤3 or 5 are not reached. But following with our principles of flexibility, it was also agreed that the lack of achievement with realistic goals also led to a treatment failure and therefore to a treatment decision. We consider that with this last statement more clinical scenarios are covered.

More specifically, we also developed some statements to assess skin clearance as nowadays there is an increasing interest in defining and incorporating into clinical practice this variable. The experts agreed that the PASI 100 response or the PASI absolute score 0 can equally be used for this purpose. However, as mentioned before, skin clearance was considered an ideal therapeutic goal.

The present project has some limitations that deserve further comments. In daily practice, there is a necessity to decide whether a treatment is able to improve the disease at a given point of time, however, we did not include a clear time frame to evaluate the treatment response because our proposal was more conceptual and already quite extensive. The same way we did not define a sufficient/minimal/acceptable improvement in an individual patient’s disease due to the same reasons. Further studies will be necessary to address these challenging and complex questions. As exposed, PASI was the selected objective outcome for the formulation of all the statements. Although it presents some limitations [29], it is the most commonly used [30] and facilitate the implementation of the statements. On the other hand, we did not generate specific recommendations on certain comorbidities like psoriatic arthritis beyond that they might modulate/influence treatment goals because they deserve a detailed analysis. The same way, we did not include specific recommendations for patients with psoriasis in specific areas involved (face, genital, nails) for the same reason. Finally, this is a qualitative study based on expert’s opinion, but taking into account the relevance of the topic and the lack of specific studies all of the exposed in this article might be very useful to stablish a treatment framework for patients with moderate to severe psoriasis.

In summary, well-defined therapeutic goals and treatment response criteria may be helpful to guide physicians in their care of patients with psoriasis. We are confident that our analyses, statements and discussions will contribute in this regard.

## Supplementary Material

Supplemental Material

## Data Availability

Data sharing is not applicable to this article as this is not a study.
