# Supplementary material for: Therapeutic goals and treatment response evaluation in moderate to severe psoriasis: an experts opinion document
Source: Ann Med. 2021 Oct 3;53(1):1727–36. doi: 10.1080/07853890.2021.1986637 (PMC8491706; doi:10.1080/07853890.2021.1986637)
Supplement: Supplemental Material [file IANN_A_1986637_SM4012.docx]

**Delphi results.**

| Nº/Delphi round | Statement | Mean (SD) | Median (p25-p75) | Min | Max | Mode | VC | ≥70% 8 or 9/ 1 or 2 | Consensus |
| --- | --- | --- | --- | --- | --- | --- | --- | --- | --- |
| 1-1^st^ | The therapeutic goal should be individualized and adapted to the characteristics of the disease and patient | 8.50 (1.44) | 9 (9-9) | 1 | 9 | 9 | 0.17 | No | Consensus (agreement) |
| 2-1^st^ | The therapeutic goal should be individualized and adapted to the characteristics of the available drugs for psoriasis | 5.42 (2.81) | 6 (2-7.25) | 1 | 9 | 9 | 0.53 | No | - |
| 2-2^nd^ |  | 5.11 (2.98) | 5 (2-8) | 1 | 9 | 2 | 0.58 | Yes | - |
| 3-1^st^ | A therapeutic goal should be established regardless of the type of drug for psoriasis | 6.92 (2.71) | 8 (6-9) | 1 | 9 | 9 | 0.40 | No | - |
| 3-2^nd^ |  | 7.13 (2.20) | 8 (6-9) | 1 | 9 | 8 | 0.31 | No | - |
| 3-3^rd^ |  | 7.44 (1.82) | 8 (7-8) | 1 | 9 | 8 | 0.25 | Yes | Consensus (agreement) |
| 4-2^nd^ | The therapeutic goal should be individualized and established in general regardless of the type of drug for psoriasis | 7.50 (2.36) | 8 (7-9) | 1 | 9 | 9 | 0.31 | Yes | - |
| 4-3^rd^ |  | 7.76 (1.44) | 8 (8-8) | 2 | 9 | 8 | 0.19 | Yes | Consensus (agreement) |
| 5-2^nd^ | The therapeutic goal should be individualized and within the limits of efficacy and safety demonstrated by the drug | 6.33 (2.78) | 7.5 (4.5-9) | 1 | 9 | 9 | 0.44 | No | - |
| 6-1^st^ | In general, in daily practice, absolute PASI is recommended to assess response to treatment | 8.44 (1.38) | 9 (8-9) | 1 | 9 | 9 | 0.17 | Yes | Consensus (agreement) |
| 7-1^st^ | In general, in daily practice, to assess response to treatment, the improvement rate is recommended over the baseline PASI | 5.00 (2.92) | 6 (1.75-8) | 1 | 9 | 1 | 0.59 | No | Inconsistent |
| 7-2^nd^ |  | 4.36 (2.66) | 3.5 (2-7) | 1 | 9 | 2 | 0.61 | Yes | Inconsistent |
| 8-2^nd^ | Sometimes, the rate of PASI improvement from baseline, complements the information from the absolute PASI and is useful to assess response to treatment | 7.86 (1.12) | 8 (7-9) | 5 | 9 | 8 | 0.14 | Yes | Inconsistent |
| 8-3^rd^ |  | 7.68 (1.21) | 8 (7-8) | 1 | 9 | 8 | 0.16 | Yes | Consensus (agreement) |
| 9-2^nd^ | In general, in daily practice, to assess response to treatment it is recommended to determine both, the rate of PASI improvement from baseline and the absolute PASI | 7.05 (2.41) | 8 (5-9) | 1 | 9 | 9 | 0.34 | Yes | Inconsistent |
| 9-3^rd^ |  | 6.62 (2.68) | 8 (6-8) | 1 | 9 | 8 | 0.41 | No | Inconsistent |
| 10-2^nd^ | In general, in daily practice, the rate of PASI improvement from baseline is preferred to absolute PASI to assess response to treatment | 2.47 (2.08) | 2 (1-2.5) | 1 | 8 | 1 | 0.84 | Yes | Consensus (disagreement) |
| 11-2^nd^ | In general, in daily practice, response to treatment could be assessed by absolute PASI improvement from baseline to assess response to treatment | 8.05 (1.67) | 9 (8-9) | 1 | 9 | 9 | 0.21 | Yes | Consensus (agreement) |
| 12-1^st^ | When establishing the therapeutic goal in moderate to severe psoriasis, it is advisable to differentiate between an ideal goal and a realistic goal | 7.64 (2.03) | 8 (7-9) | 1 | 9 | 9 | 0.27 | No | Inconsistent |
| 12-2^nd^ |  | 7.94 (1.55) | 8 (8-9) | 2 | 9 | 9 | 0.19 | Yes | Consensus (agreement) |
| 13-1^st^ | Ideal goals should include the PASI 100 response, an absolute PASI score 0 or the complete skin clearance | 8.58 (1.38) | 9 (9-9) | 1 | 9 | 9 | 0.16 | Yes | Consensus (agreement) |
| 14-1^st^ | Ideal goals should include the absence of psoriasis-related symptoms | 8.33 (1.62) | 9 (8.75-9) | 1 | 9 | 9 | 0.20 | Yes | Consensus (agreement) |
| 15-1^st^ | Ideal goals should include the absence of impact of psoriasis on patient's psychological, emotional, social and occupational domains | 8.36 (1.60) | 9 (9-9) | 1 | 9 | 9 | 0.19 | Yes | Consensus (agreement) |
| 16-1^st^ | Ideal goals should include achieving PASI 90 response | 7.75 (1.93) | 8 (7.75-9) | 1 | 9 | 9 | 0.25 | Yes | Consensus (agreement) |
| 17-1^st^ | Ideal goals should include achieving an absolute PASI score ≤ 1 | 8.06 (1.43) | 8 (8-9) | 1 | 9 | 8 | 0.18 | Yes | Consensus (agreement) |
| 18-1^st^ | Ideal goals should include achieving an absolute PASI score ≤ 3 | 6.14 (2.62) | 7 (5-8) | 1 | 9 | 7 | 0.43 | No | Inconsistent |
| 18-2^nd^ |  | 6.17 (2.55) | 7 (5.5-8) | 1 | 9 | 7 | 0.41 | No | Inconsistent |
| 19-2^nd^ | Ideal goals should include achieving an absolute PASI score ≤ 2 | 7.80 (1.47) | 8 (7.5-9) | 1 | 9 | 8 | 0.19 | Yes | Consensus (agreement) |
| 20-1^st^ | Realistic goals should include the PASI 100 response, an absolute PASI score 0 or the complete skin clearance | 5.08 (3.10) | 5.5 (2-8) | 1 | 9 | 1 | 0.62 | No | Inconsistent |
| 20-2^nd^ |  | 4.47 (2.75) | 4.5 (2-7) | 1 | 9 | 1 y 7 | 0.61 | Yes | Inconsistent |
| 21-1^st^ | Realistic goals should include achieving PASI 100 response | 4.89 (3.09) | 5 (1.75-8) | 1 | 9 | 1 | 0.64 | No | Inconsistent |
| 21-2^nd^ |  | 4.19 (2.74) | 3 (2-7) | 1 | 9 | 2 | 0.65 | Yes | Inconsistent |
| 22-1^st^ | Realistic goals should include achieving PASI 90 response | 7.28 (1.99) | 8 (6.75-9) | 1 | 9 | 8 | 0.28 | No | Inconsistent |
| 22-2^nd^ |  | 7.58 (1.98) | 8 (7.5-9) | 1 | 9 | 8 | 0.26 | Yes | Consensus (agreement) |
| 23-1^st^ | Realistic goals should include achieving PASI 75 response | 5.94 (2.68) | 6.5 (4-8) | 1 | 9 | 8 | 0.46 | No | Inconsistent |
| 23-2^nd^ |  | 6.31 (2.65) | 7 (5-9) | 1 | 9 | 9 | 0.42 | Yes | Inconsistent |
| 24-2^nd^ | In general, realistic goals should include achieving PASI 90 response, but sometimes, achieving PASI 75 response may also be realistic | 8.11 (1.60) | 9 (8-9) | 2 | 9 | 9 | 0.20 | Yes | Consensus (agreement) |
| 25-1^st^ | Realistic goals should include achieving an absolute PASI score ≤ 1 | 6.17 (2.73) | 7.5 (4-8) | 1 | 9 | 8 | 0.45 | No | Inconsistent |
| 25-2^nd^ |  | 5.53 (2.83) | 6.5 (2.5-8) | 1 | 9 | 8 | 0.51 | No | Inconsistent |
| 26-1^st^ | Realistic goals should include achieving an absolute PASI score ≤ 3 | 7.31 (2.20) | 8 (6-9) | 1 | 9 | 9 | 0.30 | No | Inconsistent |
| 26-2^nd^ |  | 7.75 (1.84) | 8 (8-9) | 1 | 9 | 8 y 9 | 0.24 | Yes | Consensus (agreement) |
| 27-2^nd^ | Realistic goals should include achieving an absolute PASI score ≤ 2 | 7.33 (2.07) | 8 (7-9) | 1 | 9 | 9 | 0.28 | Yes | Consensus (agreement) |
| 28-1^st^ | Realistic goals should include achieving an absolute PASI score ≤ 5 | 5.69 (2.64) | 6 (3-8) | 1 | 9 | 7 | 0.47 | No | Inconsistent |
| 28-2^nd^ |  | 6.0 (2.77) | 7 (3-8) | 1 | 9 | 8 | 0.46 | Yes | Inconsistent |
| 29-2^nd^ | In general, realistic goals should include achieving an absolute PASI score ≤3, but sometimes, achieving a PASI score ≤5 might also be a realistic goal | 7.44 (2.27) | 8 (7-9) | 2 | 9 | 9 | 0.30 | Yes | Inconsistent |
| 29-3^rd^ |  | 7.44 (1.65) | 8 (7-8) | 2 | 9 | 8 | 0.22 | Yes | Consensus (agreement) |
| 30-2^nd^ | In general, realistic goals should include achieving an absolute PASI score ≤3, but, with some drugs for psoriasis, achieving a PASI score ≤1 might also be possible | 7.58 (2.07) | 8 (7-9) | 1 | 9 | 9 | 0.27 | Yes | Consensus (agreement) |
| 31-1^st^ | The absolute PASI score 0 is the most appropriate measure to assess complete skin clearance | 8.22 (1.96) | 9 (9-9) | 1 | 9 | 9 | 0.24 | Yes | Consensus (agreement) |
| 32-1^st^ | The PASI 100 response is the most appropriate measure to assess complete skin clearance | 7.47 (1.83) | 8 (6.75-9) | 1 | 9 | 9 | 0.25 | No | Inconsistent |
| 32-2^nd^ |  | 7.08 (4.25) | 8 (7-9) | 1 | 9 | 8 | 0.34 | Yes | Inconsistent |
| 33-1^st^ | In daily practice, when complete skin clearance is selected to assess response to treatment, the PASI 100 response or the PASI absolute score 0 can be used | 8.56 (1.36) | 9 (9-9) | 1 | 9 | 9 | 0.16 | Yes | Consensus (agreement) |
| 34-1^st^ | In daily practice, if PASI 100 response is not achieved with a treatment (recommended doses), dose changes would be justified (treatment intensification) | 3.36 (2.44) | 2.5 (1-5.25) | 1 | 9 | 1 | 0.74 | No | Inconsistent |
| 34-2^nd^ |  | 2.94 (2.51) | 2 (1-4.5) | 1 | 8 | 1 | 0.85 | Yes | Inconsistent |
| 34-3^rd^ |  | 2.24 (1.37) | 2 (1-2.8) | 1 | 7 | 2 | 0.62 | Yes | Consensus (disagreement) |
| 35-1^st^ | In daily practice, if PASI 100 response is not achieved with a treatment (recommended doses), a treatment change would be justified | 2.78 (2.32) | 2 (1-4) | 1 | 9 | 1 | 0.85 | No | Inconsistent |
| 35-2^nd^ |  | 3.05 (2.78) | 2 (1-4) | 1 | 9 | 1 y 2 | 0.91 | Yes | Inconsistent |
| 36-1^st^ | In daily practice, if PASI 100 response is not achieved with a treatment (recommended doses), combination therapy would be justified | 4.33 (2.73) | 5 (1.75-6.25) | 1 | 9 | 1 | 0.64 | No | Inconsistent |
| 36-2^nd^ |  | 4.28 (2.91) | 4 (1-8) | 1 | 9 | 1 | 0.68 | No | Inconsistent |
| 37-1^st^ | In daily practice, if PASI 100 response is not achieved with a treatment (recommended doses), a treatment change would be justified even if it implies a cost increase | 3.08 (2.36) | 2 (1-5) | 1 | 9 | 1 | 0.78 | No | Inconsistent |
| 37-2^nd^ |  | 3.06 (2.53) | 2 (1-4) | 1 | 8 | 2 | 0.83 | Yes | Inconsistent |
| 38-1^st^ | In daily practice, if PASI 90 response is not reached with a treatment (recommended doses), dose changes would be justified (treatment intensification) | 4.92 (2.71) | 5 (2.75-7) | 1 | 9 | 9 | 0.56 | No | Inconsistent |
| 38-2^nd^ |  | 4.50 (2.61) | 4.5 (2-7) | 1 | 8 | 8 | 0.58 | No | Inconsistent |
| 39-1^st^ | In daily practice, if PASI 90 response is not reached with a treatment (recommended doses), a treatment change would be justified | 4.92 (2.76) | 5.5 (2-7) | 1 | 9 | 1 | 0.57 | No | Inconsistent |
| 39-2^nd^ |  | 5.11 (2.76) | 5.5 (2-8) | 1 | 9 | 1 y 8 | 0.54 | No | Inconsistent |
| 40-1^st^ | In daily practice, if PASI 90 response is not reached with a treatment (recommended doses), combination therapy would be justified | 5.97 (2.60) | 7 (3.75-8) | 1 | 9 | 8 | 0.44 | No | Inconsistent |
| 40-2^nd^ |  | 6.58 (2.36) | 7 (5.5-8) | 1 | 9 | 8 | 0.36 | Yes | Inconsistent |
| 40-3^rd^ |  | 5.91 (2.34) | 8 (7-8) | 1 | 9 | 8 | 0.40 | No | Inconsistent |
| 41-1^st^ | In daily practice, if PASI 90 response is not reached with a treatment (recommended doses), a treatment change would be justified even if it implies a cost increase | 4.81 (2.75) | 5 (2-7) | 1 | 9 | 7 | 0.58 | No | Inconsistent |
| 41-2^nd^ |  | 4.80 (2.77) | 5 (2-7.5) | 1 | 9 | 2 | 0.58 | No | Inconsistent |
| 42-2^nd^ | In daily practice, if PASI 75 response is not achieved with a treatment (recommended doses), dose changes would be justified (treatment intensification) | 4.83 (2.97) | 5 (2-7.5) | 1 | 9 | 1 | 0.61 | No | Inconsistent |
| 43-2^nd^ | In daily practice, if PASI 75 response is not achieved with a treatment (recommended doses), a treatment change would be justified | 7.83 (1.76) | 8 (8-9) | 1 | 9 | 8 | 0.22 | Yes | Consensus (agreement) |
| 44-2^nd^ | In daily practice, if PASI 75 response is not achieved with a treatment (recommended doses), combination therapy would be justified | 7.23 (2.13) | 8 (6-9) | 1 | 9 | 9 | 0.29 | Yes | Inconsistent |
| 44-3^rd^ |  | 6.85 (2.29) | 8 (7-8) | 1 | 9 | 8 | 0.34 | No | Inconsistent |
| 45-2^nd^ | In daily practice, if PASI 75 response is not reached with a treatment (recommended doses), a treatment change would be justified even if it implies a cost increase | 7.47 (1.86) | 8 (7-9) | 1 | 9 | 8 | 0.25 | Yes | Inconsistent |
| 45-3^rd^ |  | 7.50 (1.79) | 8 (7-8) | 1 | 9 | 8 | 0.24 | Yes | Consensus (agreement) |
| 46-2^nd^ | In daily practice, if an absolute PASI score ≤ 1 is not reached with a treatment (recommended doses), dose changes would be justified (treatment intensification) | 3.22 (2.50) | 2 (1-5) | 1 | 8 | 1 | 0.77 | No | Inconsistent |
| 47-2^nd^ | In daily practice, if an absolute PASI score ≤ 1 is not achieved with a treatment (recommended doses), a treatment change would be justified | 3.44 (2.72) | 2 (1-5) | 1 | 9 | 2 | 0.79 | Yes | Inconsistent |
| 48-2^nd^ | In daily practice, if an absolute PASI score ≤ 1 is not achieved with a treatment (recommended doses), combination therapy would be justified | 4.63 (2.91) | 4.5 (2-7.5) | 1 | 9 | 1 | 0.63 | No | Inconsistent |
| 49-2^nd^ | In daily practice, if an absolute PASI score ≤ 1 is not achieved with a treatment (recommended doses), a treatment change would be justified even if it implies a cost increase | 2.92 (2.51) | 2 (1-3) | 1 | 9 | 1 | 0.86 | Yes | Inconsistent |
| 49-3^rd^ |  | 2.15 (1.44) | 2 (1.25-2) | 1 | 9 | 2 | 0.68 | Yes | Consensus (disagreement) |
| 50-2^nd^ | In daily practice, if an absolute PASI score ≤ 3 is not reached with a treatment (recommended doses), dose changes would be justified (treatment intensification) | 5.14 (2.67) | 6 (3-8) | 1 | 9 | 8 | 0.52 | No | Inconsistent |
| 51-2^nd^ | In daily practice, if an absolute PASI score ≤ 3 is not achieved with a treatment (recommended doses), a treatment change would be justified | 5.94 (2.88) | 7 (3-8) | 1 | 9 | 8 | 0.48 | Yes | Inconsistent |
| 52-2^nd^ | In daily practice, if an absolute PASI score ≤ 3 is not achieved with a treatment (recommended doses), combination therapy would be justified | 6.80 (2.20) | 8 (6.5-8) | 1 | 9 | 8 | 0.32 | Yes | Consensus (agreement) |
| 53-2^nd^ | In daily practice, if an absolute PASI score ≤ 3 is not achieved with a treatment (recommended doses), a treatment change would be justified even if it implies a cost increase | 5.66 (2.76) | 7 (3-8) | 1 | 9 | 8 | 0.49 | Yes | Inconsistent |
| 54-2^nd^ | In daily practice, if an absolute PASI score ≤ 5 is not reached with a treatment (recommended doses), dose changes would be justified (treatment intensification) | 6.11 (2.73) | 7 (4-8.5) | 1 | 9 | 9 | 0.45 | No | Inconsistent |
| 55-2^nd^ | In daily practice, if an absolute PASI score ≤ 5 is not achieved with a treatment (recommended doses), a treatment change would be justified | 8.08 (1.5) | 8.5 (8-9) | 1 | 9 | 9 | 0.18 | Yes | Consensus (agreement) |
| 56-2^nd^ | In daily practice, if an absolute PASI score ≤ 5 is not achieved with a treatment (recommended doses), combination therapy would be justified | 7.89 (1.41) | 8 (7.5-9) | 3 | 9 | 9 | 0.18 | Yes | Consensus (agreement) |
| 57-2^nd^ | In daily practice, if an absolute PASI score ≤ 5 is not achieved with a treatment (recommended doses), a treatment change would be justified even if it implies a cost increase | 8.05 (0.89) | 8 (8-9) | 6 | 9 | 8 | 0.11 | Yes | Consensus (agreement) |
| 58-2^nd^ | In daily practice, if the realistic goal is not achieved with a treatment (recommended doses), a change of treatment strategy would be justified | 8.63 (0.64) | 9 (8-9) | 7 | 9 | 9 | 0.07 | Yes | Consensus (agreement) |
| 59-2^nd^ | In daily practice, in general, if the realistic goal is not achieved with a treatment (recommended doses), dose changes (treatment intensification) could be included as a change of treatment strategy | 6.15 (2.54) | 8 (6-8) | 1 | 9 | 8 | 0.39 | Yes | Inconsistent |
| 60-2^nd^ | In daily practice, in general, if the realistic goal is not achieved with a treatment (recommended doses), a treatment change could be included as a change of treatment strategy | 8.53 (0.61) | 9 (8-9) | 7 | 9 | 9 | 0.07 | Yes | Consensus (agreement) |
| 61-2^nd^ | In daily practice, in general, when the realistic goal not achieved with a treatment (recommended doses), combination therapy could be included as a change of treatment strategy | 7.92 (1.05) | 8 (7-9) | 5 | 9 | 8 | 0.13 | Yes | Inconsistent |
| 61-3^rd^ |  | 7.91 (0.92) | 8 (8-8) | 5 | 9 | 8 | 0.12 | Yes | Consensus (agreement) |
| 62-2^nd^ | In daily practice, if the ideal goal is achieved with a treatment (recommended doses), a change of treatment strategy would be justified | 5.97 (3.25) | 8 (2-9) | 1 | 9 | 9 | 0.54 | No | Inconsistent |
| 63-2^nd^ | In daily practice, if the ideal goal is reached with a treatment (recommended doses), dose reduction (dose down-titration and/or increasing the interval between doses) could be included as a change of treatment strategy | 8.53 (0.65) | 9 (8-9) | 6 | 9 | 9 | 0.08 | Yes | Consensus (agreement) |
| 64-2^nd^ | In daily practice, if the ideal goal is reached with a treatment (recommended doses), stopping the treatment could be included as a change of treatment strategy | 4.97 (2.77) | 5.5 (2-8) | 1 | 9 | 2 | 0.56 | No | Inconsistent |
| 65-2^nd^ | In daily practice, if the realistic goal is reached with a treatment (recommended doses), a change of treatment strategy would be justified | 3.86 (3.01) | 2 (1-7) | 1 | 9 | 1 | 0.78 | No | Inconsistent |
| 66-2^nd^ | In daily practice, if the realistic goal is achieved with a treatment (recommended doses), dose reduction (dose down-titration and/or increasing the interval between doses) could be included as a change of treatment strategy | 8.26 (1.44) | 9 (8-9) | 1 | 9 | 9 | 0.17 | Yes | Consensus (agreement) |
| 66-3^rd^ |  | 8.15 (1.26) | 8 (8-9) | 2 | 9 | 9 | 0.16 | Yes | Consensus (agreement) |
| 67-2^nd^ | In daily practice, if the realistic goal is reached with a treatment (recommended doses), stopping the treatment could be included as a change of treatment strategy | 4.4 (3.0) | 3 (2-8) | 1 | 9 | 2 | 0.69 | No | Inconsistent |
| 67-3^rd^ |  | 3.50 (2.48) | 2.5 (2-4.75) | 1 | 9 | 2 | 0.72 | No | Inconsistent |

**Abbreviations**: Min=minimum; max=maximum; SD=standard deviation; p=percentile; VC=variation coefficient.
